# Effects of Chlorogenic Acid-Enriched and Hydroxyhydroquinone-Reduced Coffee on Postprandial Fat Oxidation and Antioxidative Capacity in Healthy Men: A Randomized, Double-Blind, Placebo-Controlled, Crossover Trial

**DOI:** 10.3390/nu10040525

**Published:** 2018-04-23

**Authors:** Shun Katada, Takuya Watanabe, Tomohito Mizuno, Shinichi Kobayashi, Masao Takeshita, Noriko Osaki, Shigeru Kobayashi, Yoshihisa Katsuragi

**Affiliations:** 1 Biological Science Research Laboratories, Kao Corporation, 2-1-3 Bunka, Sumida, Tokyo 131-8501, Japan; watanabe.takuya@kao.com (T.W.); mizuno.tomohito@kao.com (T.M.); osaki.noriko@kao.com (N.O.); 2 Health Care Food Research Laboratories, Kao Corporation, 2-1-3 Bunka, Sumida, Tokyo 131-8501, Japan; kobayashi.shinichi@kao.com (S.K.); takeshita.masao@kao.com (M.T.); katsuragi.yoshihisa@kao.com (Y.K.); 3 Department of Surgery, Tokyo Rinkai Hospital, 1-4-2 Rinkai-cho, Edogawa, Tokyo 134-0086, Japan; kobayashi.shigeru1@kao.com

**Keywords:** hydroxyhydroquinone, chlorogenic acids, fat oxidation, oxidative stress

## Abstract

Chlorogenic acids (CGAs) reduce blood pressure and body fat, and enhance fat metabolism. In roasted coffee, CGAs exist together with the oxidant component hydroxyhydroquinone (HHQ). HHQ counteracts the antihypertensive effects of CGA, but its effects on CGA-induced fat oxidation (FOX) are unknown. Here we assessed the effects of CGA-enriched and HHQ-reduced coffee on FOX. Fifteen healthy male volunteers (age: 38 ± 8 years (mean ± SD); BMI: 22.4 ± 1.5 kg/m^2^) participated in this crossover study. Subjects consumed the test beverage (coffee) containing the same amount of CGA with HHQ (CGA-HHQ(+)) or without HHQ (CGA-HHQ(−)) for four weeks. Postprandial FOX and the ratio of the biological antioxidant potential (BAP) to the derivatives of reactive oxygen metabolites (d-ROMs) as an indicator of oxidative stress were assessed. After the four-week intervention, postprandial FOX and the postprandial BAP/d-ROMs ratio were significantly higher in the CGA-HHQ(−) group compared with the CGA-HHQ(+) group (4 ± 23 mg/min, group effect: *p* = 0.040; 0.27 ± 0.74, group effect: *p* = 0.007, respectively). In conclusion, reducing the amount of HHQ facilitated the postprandial FOX effects of CGA in coffee. Our findings also suggest that the mechanism underlying the inhibition of FOX by HHQ is related to postprandial oxidative stress.

## 1. Introduction

Coffee is one of the most popular beverages in the world, and its habitual intake is reported to reduce the risk of type 2 diabetes [1], heart failure [2], and hepatocellular carcinoma [3]. A recent cohort study of approximately 90,000 subjects revealed that individuals who habitually drink at least 3–4 cups of coffee a day have a reduced risk of mortality, heart disease, cerebrovascular disease, and respiratory disease [4]. Coffee contains a large amount of chlorogenic acids (CGA), which are major dietary polyphenols [5]. CGA and their metabolites have antioxidant properties [6]. In humans, CGA intake is suggested to enhance fat oxidation (FOX) [7,8] and reduce body fat [9,10].

Although CGA are particularly abundant in green coffee beans, the coffee bean roasting process produces oxidative substances such as hydroxyhydroquinone (HHQ). Self-oxidation of HHQ leads to the production of reactive oxygen species (ROS) such as superoxide anions (O_2_^−^), a source of oxidative stress [11]. Oxidative stress is due to excessive ROS from the mitochondrial respiration chain and other sources, and excess ROS production may cause mitochondrial dysfunction [12]. FOX is also affected by increased ROS generated by oxidative flux in the mitochondrial respiration chain. Animal studies revealed that HHQ likely inhibits the antihypertensive effects of CGA by promoting oxidative stress [13]. Human clinical trials also demonstrated that CGA in HHQ-reduced coffee have antihypertensive effects [14]. The effects of CGA and HHQ on postprandial FOX and oxidative stress in humans, however, are not known.

In the present study, we assessed the effects of repeated intake of coffee containing CGA with or without HHQ on postprandial FOX and oxidative stress in healthy male volunteers.

## 2. Subjects and Methods

### 2.1. Subjects

Fifteen healthy male volunteers (age: 38 ± 8 years (mean ± SD); BMI: 22.4 ± 1.5 kg/m^2^) participated in this double-blind, placebo-controlled, crossover trial. The inclusion criteria were as follows: 20–60 years of age and BMI of 20.0–29.9 (kg/m^2^). Exclusion criteria were as follows: treatment for severe disease; history of liver, renal, or heart disease; anemia; food allergies; and hypersensitivity to the test beverages. This trial was performed in accordance with the Declaration of Helsinki, and the trial plan was reviewed and approved by the local ethics committee (Kao Corporation, Tokyo, Japan). All subjects provided their written informed consent to participate in the study. The experimental protocol was registered with the University Hospital Medical Information Network (UMIN ID: 000019494).

### 2.2. Test Beverages

Two types of test beverages were prepared by Kao Corporation: CGA-enriched and HHQ-reduced coffee (CGA-HHQ(−): CGA, 428 mg; caffeine, 67 mg; HHQ, 0.08 mg/185 mL); and CGA-enriched and HHQ non-reduced coffee (CGA-HHQ(+): CGA, 382 mg; caffeine, 66 mg; HHQ, 0.57 mg/185 mL; Table 1). The CGA-HHQ(−) and CGA-HHQ(+) beverages were prepared such that they could not be distinguished by appearance or flavor. All CGA isomers are presented according to the IUPAC nomenclature [15]. The CGA-HHQ(−) beverage contained 428 mg of CGA, comprising caffeoylquinic acids (72.7%), feruloylquinic acids (18.0%), and dicaffeoylquinic acids (9.3%). The CGA-HHQ(+) beverage contained 382 mg of CGA, comprising caffeoylquinic acids (87.5%), feruloylquinic acids (8.8%), and dicaffeoylquinic acids (3.7%). The manufacturing process of the CGA-HHQ(−) beverage is nearly identical to those commonly used to produce standard coffee products, with the exception of an additional purification step to remove HHQ using active carbon. The extract derived from medium- to light-roasted coffee beans which contain higher amounts of CGA than dark-roasted coffee beans was used for CGA-HHQ(−) beverage preparation. The manufacturing process of the CGA-HHQ(+) beverage was the same as that for commercially-available canned coffee.

### 2.3. Experimental Design

The present trial had a two-phase, randomized, double-blind, placebo-controlled, crossover design. The trial comprised four measurement periods during which postprandial energy metabolism was measured 300 min after the test meal and blood samples were obtained over time. After obtaining baseline measurements, in phase I (4 weeks), subjects consumed one test beverage per day while maintaining their normal lifestyle. Measurements on day 28 of phase I were obtained between 8:00 a.m. and 2:15 p.m. After a washout period of four weeks, in phase II, the subjects consumed the other test beverage (one per day) for four weeks, again while maintaining their normal lifestyle. On day 28 (as in phase I), measurements were obtained between 8:00 a.m. and 2:15 p.m. During the trial, subjects were instructed to avoid excessive intake of coffee other than the test beverage, foods that contain a high amount of CGA (e.g., eggplant, potatoes), and foods that contain a high amount of polyphenols. Moreover, while being cautious of excessive eating and drinking, subjects were instructed to eat three meals a day and maintain their usual exercise habits. Subjects also ate prescribed meals starting with breakfast two days before each measurement day up to dinner the day before the measurement (1917 kcal/day; protein, 12E%; fat, 23E%, carbohydrates, 65E%) and were prohibited from performing strenuous exercise and drinking alcohol. The day before the measurement, subjects ate their prescribed meal by 9:00 p.m. and fasted thereafter (drinking water was allowed).

### 2.4. Indirect Calorimetry

On measurement days, subjects were prohibited from strenuous physical activity between 8:00 a.m. to 2:15 p.m., and spent the day resting and sedentary in a room with a fixed environment (room temperature, 25 °C). Using a hood-type respiratory gas analyzer (ARCO-2000, Arco System, Inc., Chiba, Japan), we assessed oxygen consumption ($\overset{\cdot }{V}O_{2}$) and carbon dioxide production (VCO_2_) during fasting [16]. At 9:00 a.m., subjects ate the test meal (555 kcal; protein, 15E%; fat, 33E%; carbohydrates, 52E%) and drank the test beverage (185 mL) within 15 min. Following this, for every 30 min starting at 9:30 a.m., postprandial $\overset{\cdot }{V}O_{2}$ and $\overset{\cdot }{V}{\mathrm{CO}}_{2}$ measurements were obtained for 15 min up to 2:15 p.m. $\overset{\cdot }{V}O_{2}$ and $\overset{\cdot }{V}{\mathrm{CO}}_{2}$ were used to determine changes in postprandial energy expenditure (EE), FOX, carbohydrate oxidation (COX), and respiratory quotient (RQ). The various parameters were calculated using the formulas [17,18]

$$\begin{array}{c} \mathrm{EE}(\mathrm{kcal})=3.9*\overset{\cdot }{V}O_{2}(L)+1.1*\overset{\cdot }{V}{\mathrm{CO}}_{2}(L)\\ \mathrm{COX}(g)=4.113*\overset{\cdot }{V}{\mathrm{CO}}_{2}(L)-2.907*\overset{\cdot }{V}O_{2}(L)-0.375*\mathrm{Protein}\;\mathrm{oxidation}(g)\\ \mathrm{FOX}(g)=1.689*\overset{\cdot }{V}O_{2}(L)-1.689*\overset{\cdot }{V}{\mathrm{CO}}_{2}(L)-0.324*\mathrm{Protein}\;\mathrm{oxidation}(g)\\ \mathrm{Protein}\;\mathrm{oxidation}(g)=[3.9*\overset{\cdot }{V}O_{2}(L)+1.1*\overset{\cdot }{V}{\mathrm{CO}}_{2}(L)]*0.125/4.32\\ \mathrm{RQ}=\overset{\cdot }{V}{\mathrm{CO}}_{2}/\overset{\cdot }{V}O_{2} \end{array}$$

### 2.5. Dietary Records

Subjects recorded details of their meals from three to five days before measurement days during each measurement period. Using the dietary records, registered dieticians performed nutritional value calculations for total energy intake, protein intake, fat intake, and carbohydrate intake using nutrition calculation software (Healthy Maker Pro, Mushroomsoft Co., Ltd., Okayama, Japan) that conforms to the Standard Tables of Food Composition in Japan (fifth revised edition; Table 2).

### 2.6. Blood Analysis

On each measurement day, blood was drawn at 8:15 a.m. under fasting conditions, and at 30 min, 60 min, 120 min, and 240 min after eating and drinking the test meal and beverage. Fasting serum low-density lipoprotein cholesterol (LDL-C), high-density lipoprotein cholesterol (HDL-C), total cholesterol (Total-C), triglyceride (TG), γ-glutamyltransferase (γ-GTP), and non-esterified fatty acids (NEFA) were measured using enzymatic methods. Fasting blood glucose was measured using a blood glucose meter (ACCU-CHEK Aviva; Roche Diagnostics K.K., Tokyo, Japan). Plasma biological antioxidant potential (BAP) and derivatives of reactive oxygen metabolites (d-ROMs) were measured over time using the FRAS4 analytical system (Wismerll Co., Ltd., Tokyo, Japan). Measurements of fasting serum LDL-C, HDL-C, Total-C, TG, γ-GTP, and NEFA were performed by LSI Medience Corporation (Tokyo, Japan).

### 2.7. Statistical Analysis

Total area under the curve (tAUC) was calculated using the trapezoid rule. All crossover data for the two groups were compared using the paired *t*-test (two-sided, α = 0.05). Sample size was calculated based on a power analysis using preliminary data with an α error of 0.05 and a β error of 0.2. A study group of 15 subjects was required for a power of 80% and allowing for 20% drop-out. A mixed model analysis of variance (ANOVA) was used to assess group, time, and group × time interactions, with postprandial changes in EE, FOX, RQ, and the BAP/d-ROMs ratio set as the main effects. All statistical analyses were performed with IBM SPSS Statistics for Windows, ver.19.0 (SPSS Inc., Chicago, IL, USA).

## 3. Results

### 3.1. Subjects

Fifteen healthy subjects participated in and completed the trial. The subject characteristics are summarized in Table 3, and all parameters were within the normal range. Nutritional value calculations based on total energy intake, protein intake, fat intake, and carbohydrate intake determined from dietary records taken three to five days before the measurement days revealed no significant differences between the CGA-HHQ(−) and CGA-HHQ(+) groups after the intervention (Table 2). Intake of prescribed meals from two days before the measurement days and test beverages during the intervention period was compiled from diaries and compliance was 100% for both groups. The subject characteristics did not differ significantly between the CGA-HHQ(−) and CGA-HHQ(+) groups (Table 3).

### 3.2. Postprandial Energy Metabolism

Table 4 shows changes in EE, FOX, and RQ over the course of 300 min after eating the test meal following the four-week intervention in the CGA-HHQ(−) and CGA-HHQ(+) groups. Postprandial EE was similar between the two groups. Postprandial FOX was significantly higher in the CGA-HHQ(−) group than in the CGA-HHQ(+) group (the size of the difference: 4 ± 23 mg/min, two-way ANOVA, group effect: *p* = 0.040). Postprandial RQ was significantly lower in the CGA-HHQ(−) group than in the CGA-HHQ(+) group (the size of the difference: −0.02 ± 0.05, two-way ANOVA, group effect: *p* < 0.001). Following the four-week intervention and 300 min after eating the test meal, total EE (tAUC) was 370 ± 35 kcal/300 min in the CGA-HHQ(−) group and 372 ± 47 kcal/300 min in the CGA-HHQ(+) group (paired *t*-test, *p* = 0.903); total FOX (tAUC) was 15.8 ± 5.8 g/300 min in the CGA-HHQ(−) group and 16.6 ± 4.8 g/300 min in the CGA-HHQ(+) group (paired *t*-test, *p* = 0.740); total COX (tAUC) was 41.3 ± 8.8 g/300 min in the CGA-HHQ(−) group and 39.9 ± 8.1 g/300 min in the CGA-HHQ(+) group (paired *t*-test, *p* = 0.698); and postprandial mean RQ was 0.845 ± 0.035 in the CGA-HHQ(−) group and 0.844 ± 0.028 in the CGA-HHQ(+) group (paired *t*-test, *p* = 0.695), with no significant differences in these parameters between the two groups.

### 3.3. Blood Analysis

Analysis of oxidative stress in the blood revealed a significantly higher postprandial BAP/d-ROMs ratio in the CGA-HHQ(−) group than in the CGA-HHQ(+) group (the size of the difference: 0.27 ± 0.74, two-way ANOVA, group effect: *p* = 0.007, Table 5). Analysis of blood parameters under fasting conditions is shown in Table 6.

## 4. Discussion

The present study evaluated the effects of consumption of coffee beverages with the same CGA and caffeine content, but a different HHQ content, and found that CGA-enriched and HHQ-reduced coffee enhanced postprandial FOX. Further, consuming CGA-enriched and HHQ-reduced coffee also affects the postprandial BAP/d-ROMs ratio. A recent concise report indicated that reducing HHQ in coffee affects the increase in FOX after CGA consumption [19]. Consistent with this finding, the results of the present study suggest that concomitant HHQ consumption with CGA interferes with the CGA-induced increase in FOX.

The fact that HHQ increases oxidative stress raises the possibility that oxidative stress is involved in the suppressive effects of HHQ on the increase in FOX after CGA consumption. Oxidative stress is involved in the pathogenesis of various diseases, such as cardiovascular disease [20,21,22]. Levels of the antioxidative marker BAP decrease with aging, whereas the levels of d-ROMs, a marker of oxidative stress, increase with aging [23]. Recent studies reported that oxidative stress is also involved in obesity [24,25,26]. Indicators of oxidative stress are positively correlated with visceral fat area [25], whereas BAP is negatively correlated with corrected BMI [26]. Continuous administration of CGA to C57BL/6J mice for two weeks reduced the expression of sterol regulatory element-binding protein 1c (SREBP-1c), a regulator of fatty acid synthesis, and its related factors [27]. Reducing SREBP-1c expression decreases the malonyl-CoA concentration in the liver, which alleviates the inhibition of carnitine palmitoyltransferase I (CPT-1), the rate-limiting enzyme of fatty acid oxidation, by malonyl-CoA, likely leading to enhanced FOX [28]. Recent cell-based experiments revealed that CPT-1 activity is markedly reduced in the presence of oxidative stress [29]. Considering that HHQ is a source of oxidative stress, CPT-1 may be affected by HHQ-related oxidation, thereby altering the CGA enhancement of FOX. Green coffee bean extracts and CGA have anti-obesity effects in rodents [30,31]. Moreover, in humans, consuming HHQ-reduced coffee increases postprandial FOX [7,8] and reduces body weight and body fat [9] compared to placebo coffee lacking CGA. In the present study, the CGA-HHQ(−) group exhibited increased postprandial FOX and a higher postprandial BAP/d-ROMs ratio (i.e., less oxidative stress) compared with the CGA-HHQ(+) group. These findings suggest that reducing the HHQ content in coffee may be necessary for the CGA effects to induce FOX related to CPT-1 activity.

Nitric oxide (NO) dynamics may also be involved in the present findings. Doulias et al. reported that nitrosylation of Cys238 on very long chain acyl-CoA dehydrogenase, a FOX-related enzyme, regulates the catalytic efficiency of the enzyme [32]. If oxidative stress derived from HHQ reduces the bioavailability of NO, this could lead to the inhibition of NO-mediated FOX regulation by very long chain acyl-CoA dehydrogenase.

It has been known that caffeine stimulates EE and FOX [33,34]. Although the CGA-HHQ(−) and CGA-HHQ(+) beverages used in the present study contained approximately the same amount of caffeine (CGA-HHQ(−): 67 mg caffeine/185 mL; CGA-HHQ(+): 66 mg caffeine/185 mL), we cannot exclude the potential synergistic effects of CGA and caffeine on the increase in postprandial FOX observed in this study. Another limitation of this study is that we did not evaluate whether inhibition of the increase in FOX after CGA consumption by HHQ is limited to after meals. Although d-ROMs assay frequently used to assess oxidative stress [35,36], additional biomarker to assess oxidative stress is also needed to determine the impact of postprandial oxidative stress on fat oxidation. Moreover, with respect to the small sample size in this study and the involvement of HHQ in the body weight and fat-reducing effects of CGA, further studies using larger sample sizes are needed.

## 5. Conclusions

In conclusion, we demonstrated that four weeks’ ingestion of CGA-enriched and HHQ-reduced coffee increases postprandial FOX compared with ingestion of HHQ non-reduced coffee. Moreover, the mechanism by which HHQ suppresses the increase in FOX after CGA consumption is likely related to oxidative stress. Our findings suggest that reducing HHQ in coffee is an important process that permits coffee drinkers to obtain the maximal health benefits of CGA.

## Figures and Tables

**Table 1:** Test beverage composition

	CGA-HHQ(−) Beverage	CGA-HHQ(+) Beverage
Brix (%)	1.9	2.1
pH	5.8	5.6
CGA (mg/185 mL)	428	382
Caffeine (mg/185 mL)	67	66
HHQ (mg/185 mL)	0.08	0.57

CGA, chlorogenic acids; HHQ, hydroxyhydroquinone.

**Table 2:** Dietary records

	CGA(+)	
	HHQ(−)	HHQ(+)
Energy intake (kcal/day)	2014 ± 392	1948 ± 354
Protein intake (g/day)	71.0 ± 15.5	70.5 ± 15.5
Fat intake (g/day)	71.3 ± 19.5	66.4 ± 16.1
Carbohydrate intake (g/day)	248.9 ± 49.9	237.8 ± 45.8

No significant differences were detected between groups. Data are expressed as mean ± SD, *n* = 15.

**Table 3:** Subject characteristics

	CGA(+)	
	HHQ(−)	HHQ(+)
Age (years)	38 ± 8	
Height (cm)	174.6 ± 4.5	
Weight (kg)	68.2 ± 5.8	68.4 ± 5.6
BMI (kg/m^2^)	22.4 ± 1.6	22.4 ± 1.5
Body fat (%)	19.7 ± 3.7	19.8 ± 2.9
SBP (mmHg)	127 ± 11	125 ± 9
DBP (mmHg)	75 ± 9	72 ± 9
Glucose (mg/dL)	94 ± 7	95 ± 8
LDL-C (mg/dL)	109 ± 18	109 ± 21
HDL-C (mg/dL)	57 ± 12	58 ± 12
Total-C (mg/dL)	188 ± 22	189 ± 23
TG (mg/dL)	95 ± 40	94 ± 28
NEFA (mEq/L)	0.40 ± 0.13	0.35 ± 0.08
γ-GTP (U/L)	28 ± 16	28 ± 18
BAP/d-ROMs	6.8 ± 0.6	6.9 ± 0.8

No significant differences were detected between groups. Data are expressed as mean ± SD, *n* = 15. SBP, systolic blood pressure; DBP, diastolic blood pressure; LDL-C, low-density lipoprotein cholesterol; HDL-C, high-density lipoprotein cholesterol; Total-C, total cholesterol; TG, triglyceride; NEFA, non-esterified fatty acids; γ-GTP, γ-glutamyltransferase; BAP, biological antioxidant potential; d-ROMs, derivatives of reactive oxygen metabolites.

**Table 4:** Changes in EE, FOX, and RQ up to 300 min after the test meal following the four-week intervention

	Group	Time (min)										Mean	*p* Value		
		30	60	90	120	150	180	210	240	270	300		Group	Time	Group × Time
EE, cal/min	CGA-HHQ(+)	168 ± 94	248 ± 100	250 ± 73	234 ± 101	215 ± 79	179 ± 68	95 ± 63	88 ± 108	100 ± 67	52 ± 54	163 ± 57	0.536	<0.001	0.728
	CGA-HHQ(−)	177 ± 65	207 ± 103	261 ± 84	244 ± 53	233 ± 90	141 ± 79	84 ± 83	97 ± 60	75 ± 75	59 ± 106	158 ± 58			
FOX mg/min	CGA-HHQ(+)	17 ± 21	8 ± 21	−4 ± 13	1 ± 24	−5 ± 22	−7 ± 22	−3 ± 19	15 ± 20	22 ± 24	21 ± 22	7 ± 16	0.040	<0.001	0.155
	CGA-HHQ(−)	15 ± 16	−3 ± 20	−2 ± 18	1 ± 18	−4 ± 23	0 ± 24	12 ± 18	28 ± 19	26 ± 20	32 ± 28	11± 17			
RQ	CGA-HHQ(+)	−0.02 ± 0.05	0.01 ± 0.04	0.03 ± 0.03	0.02 ± 0.06	0.04 ± 0.06	0.04 ± 0.05	0.02 ± 0.05	−0.03 ± 0.05	−0.04 ± 0.06	−0.04 ± 0.06	0.00 ± 0.04	<0.001	<0.001	0.201
	CGA-HHQ(−)	−0.02 ± 0.04	0.02 ± 0.05	0.02 ± 0.04	0.01 ± 0.04	0.03 ± 0.06	0.01 ± 0.06	−0.02 ± 0.05	−0.06 ± 0.05	−0.06 ± 0.05	−0.07 ± 0.07	−0.01 ± 0.04			

Data are expressed as mean ± SD, *n* = 15. EE, energy expenditure; FOX, fat oxidation; RQ, respiratory quotient.

**Table 5:** Changes in the BAP/d-ROMs ratio up to 240 min after the test meal following the four-week intervention

	Group	Time (min)				Mean	*p* Value		
		30	60	120	240		Group	Time	Group × Time
BAP/d-ROMs	CGA-HHQ(+)	−0.12 ± 0.83	−0.07 ± 0.74	−0.06 ± 0.68	−0.23 ± 0.63	−0.12 ± 0.64	0.007	0.311	0.806
	CGA-HHQ(−)	0.12 ± 0.55	0.36 ± 0.55	0.12 ± 0.55	0.00 ± 0.34	0.15 ± 0.38			

Data are expressed as mean ± SD, *n* = 15.

**Table 6:** Blood parameters under fasting conditions after four-week intervention

	CGA(+)	
	HHQ(−)	HHQ(+)
Glucose (mg/dL)	94 ± 7	91 ± 7
Insulin (μU/mL)	3.8 ± 1.6	4.0 ± 1.9
LDL-C (mg/dL)	106 ± 17	106 ± 26
HDL-C (mg/dL)	60 ± 14	56 ± 13
Total-C (mg/dL)	187 ± 23	184 ± 35
TG (mg/dL)	92 ± 30	101 ± 41
NEFA (mEq/L)	0.37 ± 0.10	0.36 ± 0.09
γ-GTP (U/L)	25 ± 14	28 ± 18
BAP/d-ROMs	6.7 ± 0.9	6.7 ± 0.9

No significant differences were detected between groups. Data are expressed as mean ± SD, *n* = 15.

## Abbreviations

CGA	chlorogenic acids
HHQ	hydroxyhydroquinone
$\overset{\cdot }{V}$O_2_	oxygen consumption
$\overset{\cdot }{V}$CO_2_	carbon dioxide production
EE	energy expenditure
FOX	fat oxidation
COX	carbohydrate oxidation
RQ	respiratory quotient
BAP	biological antioxidant potential
d-ROMs	derivatives of reactive oxygen metabolites
SREBP-1c	sterol regulatory element-binding protein 1c
CPT-1	carnitine palmitoyltransferase I
NO	nitric oxide
